# A novel nanobody-heavy chain antibody against Angiopoietin-like protein 3 reduces plasma lipids and relieves nonalcoholic fatty liver disease

**DOI:** 10.1186/s12951-022-01456-z

**Published:** 2022-05-19

**Authors:** Xiaozhi Hu, Jiajun Fan, Qianqian Ma, Lei Han, Zhonglian Cao, Caili Xu, Jingyun Luan, Guangjun Jing, Yanyang Nan, Tao Wu, Yuting Zhang, Hanqi Wang, Yuanzhen Zhang, Dianwen Ju

**Affiliations:** 1 School of Pharmacy & Minhang Hospital, Shanghai Engineering Research Center of Immunotherapeutic, Fudan University, Shanghai, 201203 China; 2 National Children’s Medical Center, Children’s Hospital of Fudan University, Shanghai, 201203 China; 3 Ben May Department of Cancer Research, The University of Chicago, Chicago, IL 60615 USA

**Keywords:** ANGPTL3, Nanobody, Lipid-reducing, Hypercholesterolemia, NAFLD

## Abstract

**Background:**

Nonalcoholic fatty liver disease (NAFLD) is a metabolic disease mainly on account of hypercholesterolemia and may progress to cirrhosis and hepatocellular carcinoma. The discovery of effective therapy for NAFLD is an essential unmet need. Angiopoietin-like protein 3 (ANGPTL3), a critical lipid metabolism regulator, resulted in increased blood lipids and was elevated in NAFLD. Here, we developed a nanobody-heavy chain antibody (VHH-Fc) to inhibit ANGPTL3 for NAFLD treatment.

**Results:**

In this study, we retrieved an anti-ANGPTL3 VHH and Fc fusion protein, C44-Fc, which exhibited high affinities to ANGPTL3 proteins and rescued ANGPLT3-mediated inhibition of lipoprotein lipase (LPL) activity. The C44-Fc bound a distinctive epitope within ANGPTL3 when compared with the approved evinacumab, and showed higher expression yield. Meanwhile, C44-Fc had significant reduction of the triglyceride (~ 44.2%), total cholesterol (~ 36.6%) and LDL-cholesterol (~ 54.4%) in hypercholesterolemic mice and ameliorated hepatic lipid accumulation and liver injury in NAFLD mice model.

**Conclusions:**

We discovered a VHH-Fc fusion protein with high affinity to ANGPTL3, strong stability and also alleviated the progression of NAFLD, which might offer a promising therapy for NAFLD.

**Graphical Abstract:**

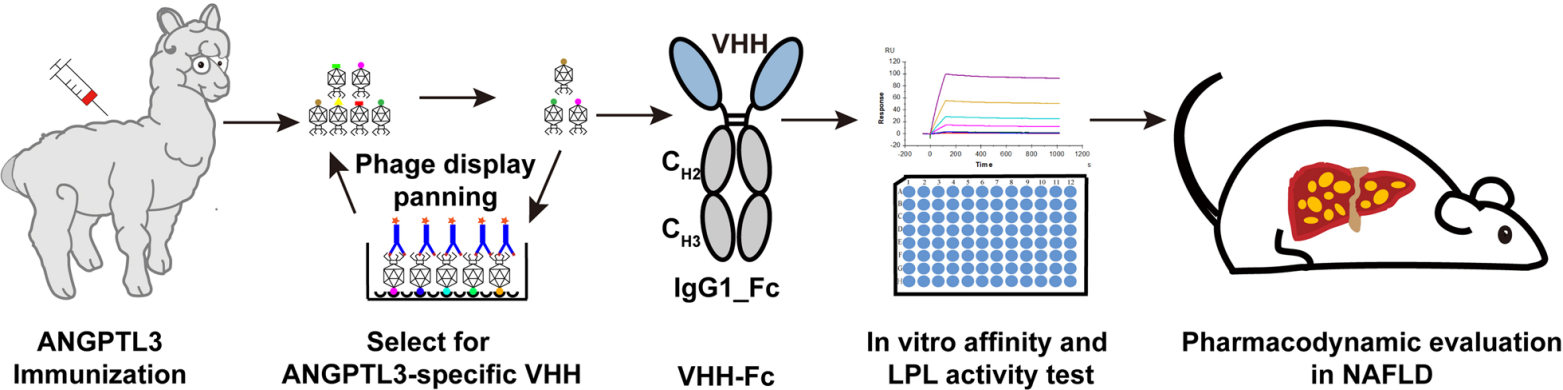

**Supplementary Information:**

The online version contains supplementary material available at 10.1186/s12951-022-01456-z.

## Background

Hypercholesterolemia, accompanied by increased total cholesterol (TC) including low-density lipoprotein (LDL), shows positive correlation with nonalcoholic fatty liver disease (NAFLD) [1, 2]. As one of the major metabolic organs, liver is closely regulated in response to hypercholesterolemia, undergoes steatosis and then evolves into NAFLD [3]. NAFLD is the most prevalent liver disease and characterized by abnormal hepatic triglyceride (TG) and cholesterol accumulations. Its pathology ranges from nonalcoholic steatohepatitis to cirrhosis and even progresses to hepatocellular carcinoma [4]. Although there have been advances in lipid-lowering therapy including peroxisome proliferation activating receptors (PPAR)-α/γ agonists and statins, NAFLD still afflicted 1.7 billion population worldwide [5–7]. Therefore, the novel therapeutics for NAFLD are imminently craved.

Angiopoietin-like protein 3 (ANGPTL3), as a lipometabolic regulator, was a secretory protein and primarily expressed in the liver [8]. It could be cleaved into a coiled-coil domain (CCD) and a fibrinogen-like domain (FLD). The CCD and the full-length of ANGPTL3 all could inhibit the enzyme activity of lipoprotein lipase (LPL) as well as endothelial lipase (EL) [8–11]. The individuals with ANGPTL3-function deficiency existed natively with low plasma TG and TC levels and displayed no clinical risks [12, 13]. It had been well documented that the high-cholesterol diet could elevate ANGPTL3 expression levels as a consequence of liver X receptors (LXRs) activation, which aggravated hypertriglyceridemia [14, 15]. Besides, ANGPTL3 had increased expression levels in the liver of NAFLD patients, which resulted in elevated circulating ANGPTL3, indicating ANGPTL3 may be a critical target in NAFLD [16]. The ANGPTL3 inhibitors could efficiently alleviate plasma TG and TC levels in hypercholesterolemic patients and relieve the progress of atherosclerosis [17, 18].

Up to now, three diverse blockade tactics against ANGPTL3 have been validated, including antisense oligonucleotide (ASO, IONIS-ANGPTL3-LRx), the CRISPR/Cas9 gene knockout technology and the monoclonal antibody [19–21]. Unfortunately, some of them have severe limitations and their druggability remain controversial. As was reported previously, the ASO-based drugs were easily degradable and the CRISPR/Cas9 gene knockout technology had off-target effects and ethical limitations [22, 23]. In addition to conventional antibody, nanobody is a camelids single domain antibody (VHH) at the nanometer scale, which has various advantages over the classical immunoglobulin gamma (IgG) [24–26]. It could be stably expressed with high yield in mammalian cell, yeast and even bacteria [27, 28]. It has efficient penetration even through the blood–brain barrier due to nanometer scale, superior thermostability and chemostability, preeminent specificity and binding-ability to antigen [29–34]. Lately, the first nanobody drug, a bivalent VHHs fusion protein (caplacizumab) was approved to treat thrombotic thrombocytopenic purpura by the European Commission [35]. Thus, VHH fusion protein blocking ANGPTL3 may be a promising treatment for hypercholesterolemic and NAFLD.

**Fig. 1:**
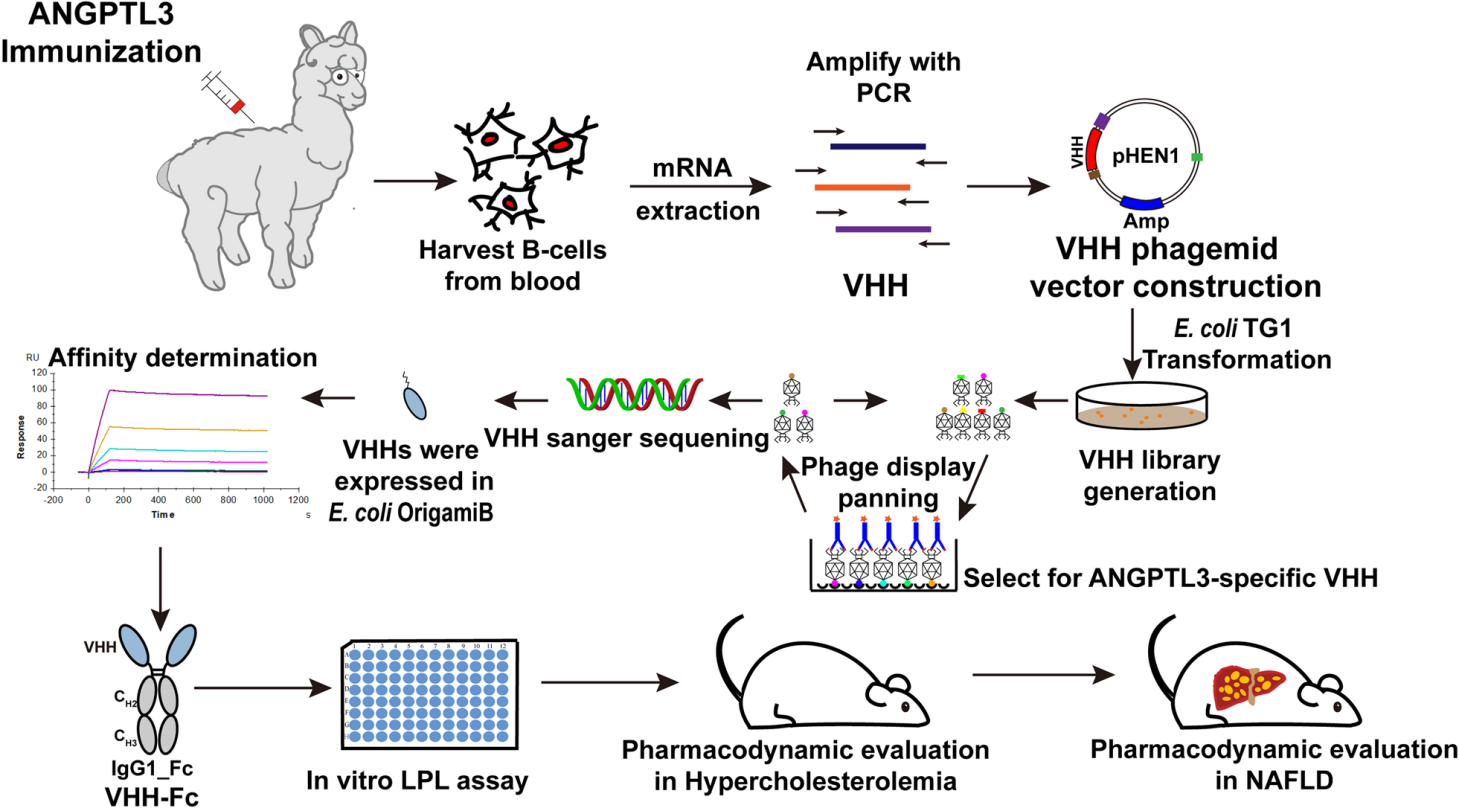
Schematic presentation of the whole experiment. The whole experimental procedure includes immunization with ANGPTL3, B-cells separation, VHH library construction, phage-display to select ANGPTL3-specific VHH, sanger sequencing, VHH and VHH-Fc fusion proteins expression, affinity determination, in vitro LPL assay and pharmacodynamic evaluation in hypercholesterolemic and NAFLD mice model

In this manuscript, we aim to discover the therapeutic effect of VHHs fusion protein against ANGPTL3 on NAFLD. After immunizing an alpaca with human ANGPTL3 (S17-K170) (hANGPTL3), we retrieved the VHH binding to the CCD of ANGPTL3 with high affinity, which were then fused to human IgG1-Fc to establish the fusion protein. Our results indicated that the VHH-Fc fusion protein could block ANGPTL3-mediated suppression of LPL activity in vitro, reduce serum lipid levels and ameliorated hepatic lipid accumulation and liver injury in NAFLD mice (Fig. 1).

## Results

### Construction of the VHHs phage library

**Fig. 2:**
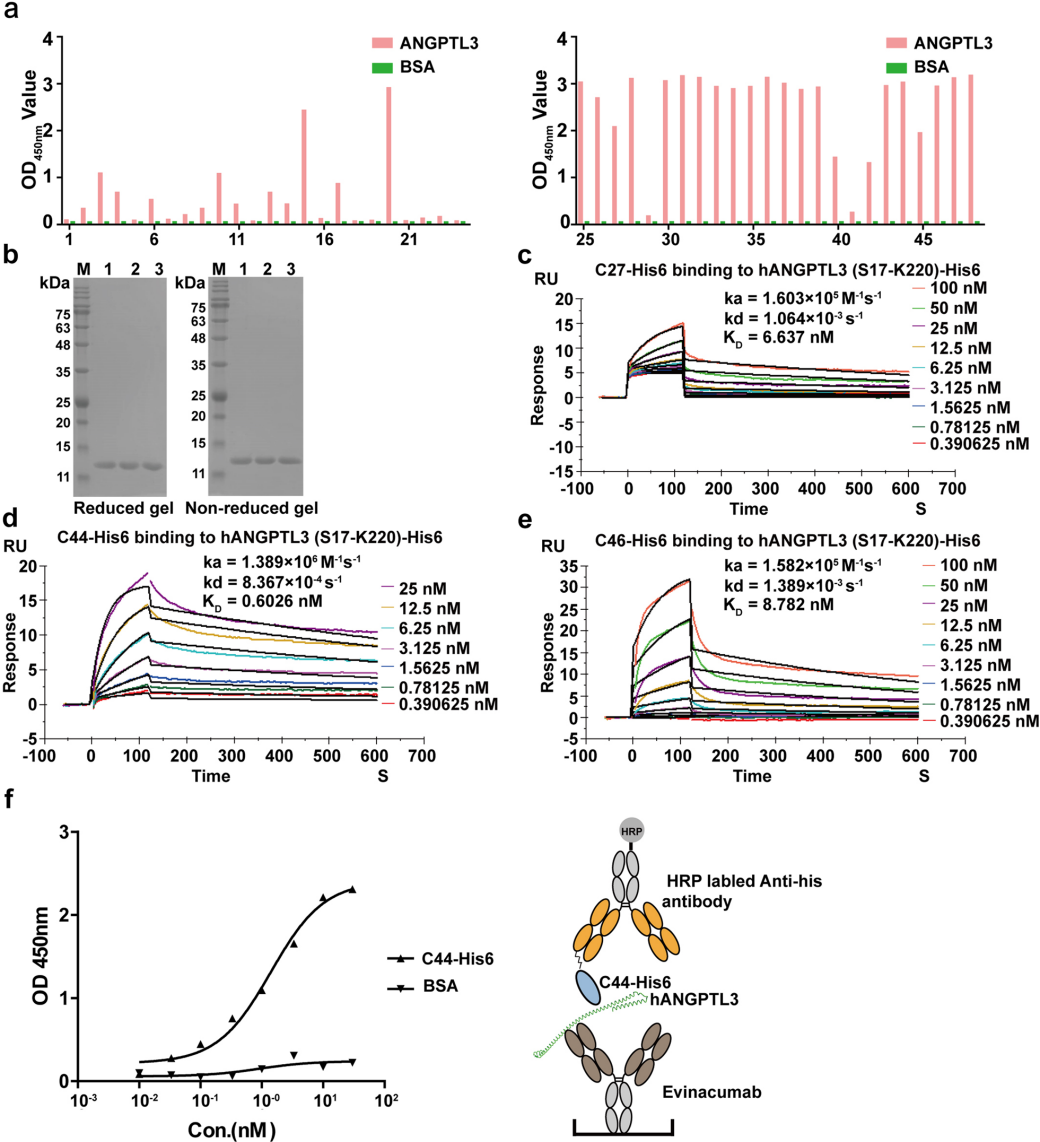
The affinity test of anti-hANGPTL3-CCD VHHs and epitope binning assay. **a** Identification of ANGPTL3 specific VHHs from 48 clones specifically binding with hANGPTL3 after three rounds bio-panning. **b** The SDS-PAGE analysis of the C27-His6, C44-His6 and C46-His6. M: Maker; lane 1: C27-His6; lane 2: C44-His6; lane 3: C46-His6. **c**–**e** The affinity of C27-His6, C44-His6 and C46-His6 binding to hANGPTL3 (S17-P220)-His6. **f** The assay for hANGPTL3-binding epitopes of the C44-His6 was performed against evinacumab by the dual antibody sandwich ELISA. The horizontal and vertical axes represent the concentrations of the C44-His6 and the OD450 value

After immunizing an alpaca for four rounds of hANGPTL3 (S17-K170), the serological ELISA was performed with 200 ng of hANGPTL3 (S17-K170) antigen/well. As shown in Additional file 1: Fig. S1, the titer of serological antibody specific to the hANGPTL3 (S17-K170) was 1:64,000. The immune VHH library was developed subsequently and the library size was approximate 5 × 10^9^ by counting the number of transformants of *E. coli* TG1 with ten-fold serial dilution. Furthermore, the VHH genes insertion rate of library were measured by PCR, which was determined to be 97.91% through randomly picking 48 clones (Additional file 1: Fig. S2). After three rounds of bio-panning using hANGPTL3 (S17-220P)-His6, 33 candidate clones specifically binding with hANGPTL3 were identified (Fig. 2a). The 33 positive clones were sequenced and their CDR3 domains were analyzed, and we obtained three nanobodies (C27, C44 and C46) with unique CDR3 domains.

### Expression of nanobody and kinetics screening

The three candidate nanobodies with 6 × His tag at C terminal (C27-His6, C44-His6 and C46-His6) were subsequently expressed in *E. coli* Origami B(DE3) strain and successfully retrieved from *E. coli* cytoplasm through ultrasonic degradation (Fig. 2b). C44-His6 showed the highest affinity to hANGPTL3 (S17-P220)-His6 at 0.6026 nM (Fig. 2c). The other two clones (C27-His6 and C46-His6) rapidly dissociated after binding to hANGPTL3 (S17-P220)-His6 (Fig. 2d, e). The epitope binning test of the C44 was performed against the positive control evinacumab by a dual antibody sandwich ELISA [48]. Evinacumab coated on a plate was employed to capture hANGPTL3 (S17-K170)-Fc protein, the C44-His6 could bind other epitopes on hANGPTL3 (S17-K170)-Fc (Fig. 2f), which suggested C44-His6 had different binding epitopes when compared with the evinacumab.

### Expression of VHH-Fc and affinity test

**Fig. 3:**
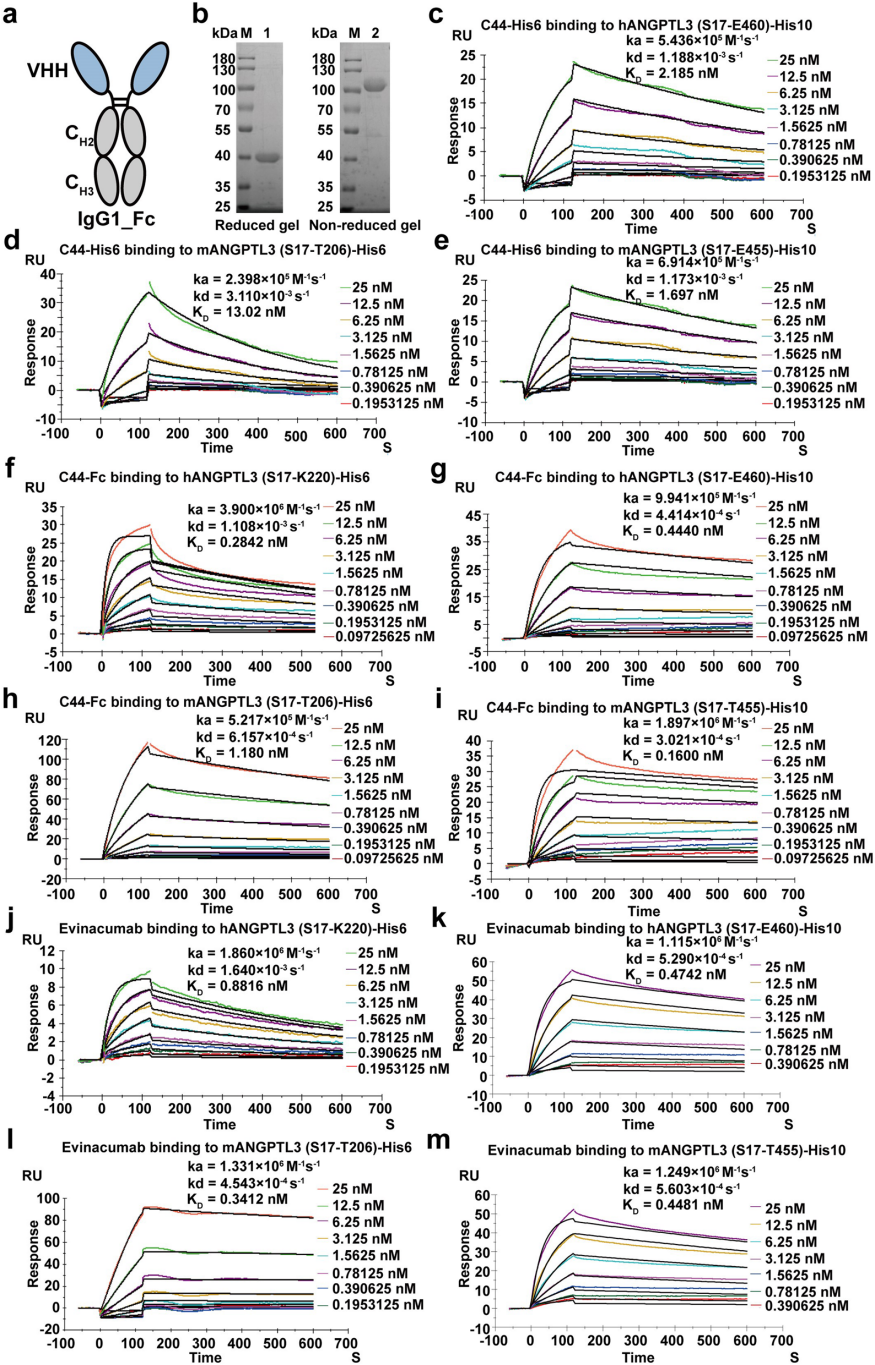
The affinity test of C44-Fc with human and mouse ANGPTL3 proteins. **a** Diagram of the C44 fused with human IgG1 Fc fragments. **b** The SDS-PAGE analysis of the C44-Fc. M: Maker; lane 1: SDS-PAGE analysis of C44-Fc in reduced gel; lane 2: SDS-PAGE analysis of C44-Fc in non-reduced gel. **c**, **g** and **k** The affinity of C44-His6, C44-Fc and evinacumab binding to hANGPTL3 (S17-E460)-His10. **d**, **h** and **l** The affinity of C44-His6, C44-Fc and evinacumab binding to mANGPTL3 (S17-T206)-His6. **e**, **i** and **m** The affinity of C44-His6, C44-Fc and evinacumab binding to mANGPTL3 (S17-T455)-His10. **f** and **j** The affinity of C44-Fc and evinacumab binding to hANGPTL3 (S17-P220)-His6

To extend the half-life of nanobody, C44 was fused to the Fc domain of human IgG1 to construct the alpaca-human chimeric antibody C44-Fc (Fig. 3a). The C44-Fc was then expressed in ExpiCHO cells using a commercial pTT5 vector. The SDS-PAGE results in Fig. 3b demonstrated that the C44-Fc fusion protein was appropriately produced with high purity (78 kDa in non-reduced PAGE and 39 kDa in reduced PAGE). As both the CCD and the full-length of ANGPTL3 proteins could inhibit the LPL activity, we also investigated the affinity of C44-His6 and C44-Fc to the human or mouse ANGPTL3 domains as well as CCD fragments. C44-Fc had excellent affinity to mANGPTL3 (S17-T455)-His10 at 0.1600 nM, hANGPTL3 (S17-P220)-His6 at 0.2842 nM, hANGPTL3 (S17-E460)-His10 at 0.4440 nM, mANGPTL3 (S17-T206)-His6 at 1.180 nM and (Fig. 3c–m), displaying better affinities comparing with the monomeric VHH C44-His6. Compared with evinacumab, C44-Fc displayed ~ 3 times higher affinities to the CCD fragment of hANGPTL3 and mANGPTL3, but a lower affinity to the CCD fragment of mANGPTL3. As a result, C44-Fc could crossly bind to human and mouse ANGPTL3 proteins as well as CCD fragment at high affinities.

### C44-Fc shown strong stability and inhibitory effect on ANGPTL3

**Fig. 4:**
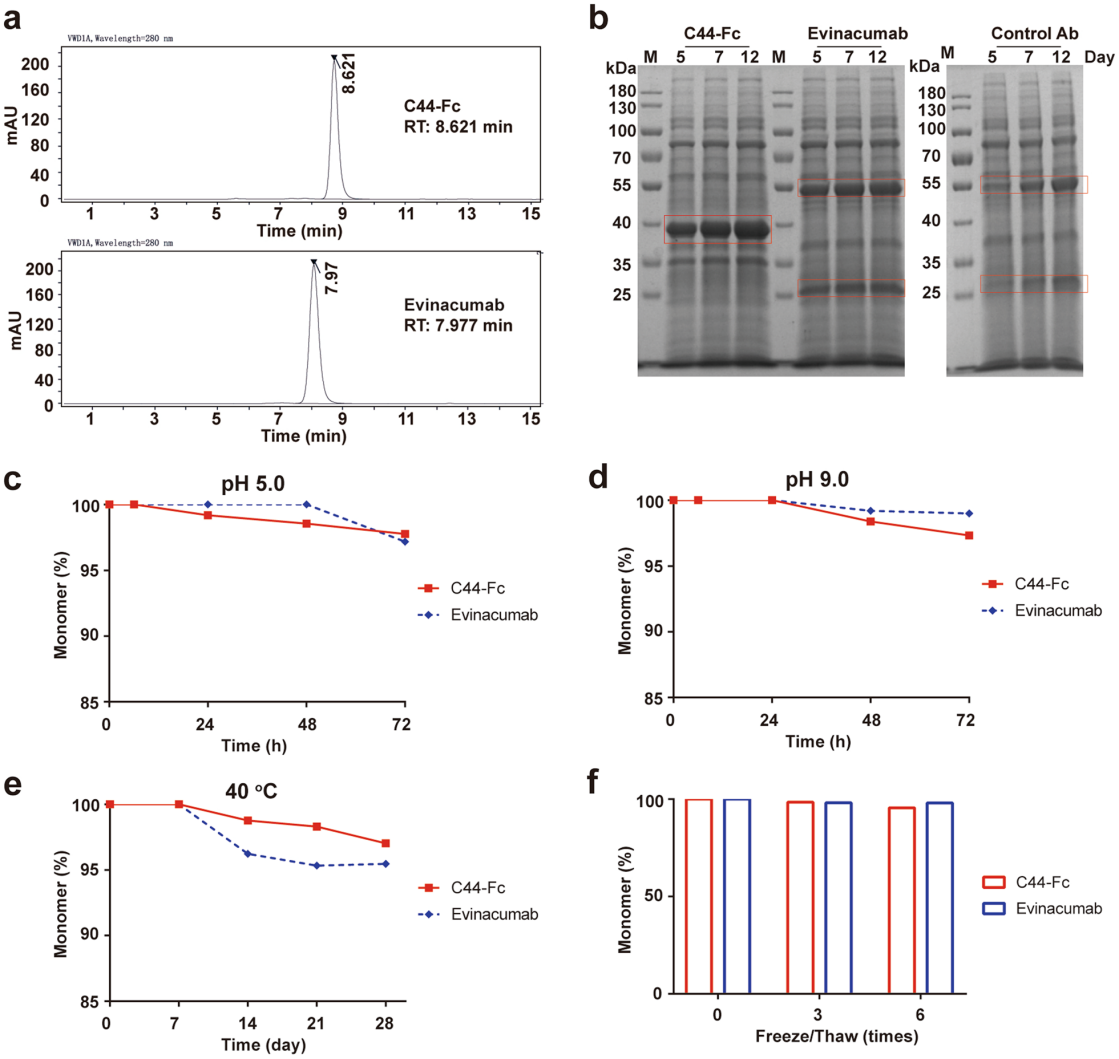
The biophysical stability evaluation of C44-Fc in vitro. **a** SEC-HPLC profile of C44-Fc and evinacumab. **b** Time course analysis of C44-Fc expression levels in ExpiCHO cells. The culture supernatants of ExpiCHO cells transiently transfected with C44-Fc, evinacumab and control Ab expression plasmid were collected on days 5–12. **c** and **d** SEC-HPLC analysis of C44-Fc and evinacumab at low or high pH. C44-Fc and evinacumab were preserved at pH 5.0 or pH 9.0 and 40 ℃ for 72 h. **e** SEC-HPLC analysis of C44-Fc and evinacumab at 40 ℃ for 28 days. **f** The freeze–thaw stability analysis of C44-Fc and evinacumab by SEC-HPLC

At first, the molecular size variations of recombinant C44-Fc protein were analyzed by SEC-HPLC. From the Fig. 4a, the C44-Fc had 100 percent of the main peak purity without aggregation or fragment and its molecular size was smaller than evinacumab. Thereafter, the yield and stability of C44-Fc were further analyzed. In the industry-standard ExpiCHO cell system, the fusion proteins C44-Fc reached its expression level of 500 mg/L, which was roughly twice as much as evinacumab (Fig. 4b). We applied several conditions of induced degradation to test biophysical stability by SEC-HPLC, including low pH (~ 5.0), high pH (~ 9.0), high temperature of 40 °C and freeze–thaw. As shown in Fig. 4c–f, all of the main peak were over 97 percentages, indicating that the stability of C44-Fc protein was strong and comparable to evinacumab.

**Fig. 5:**
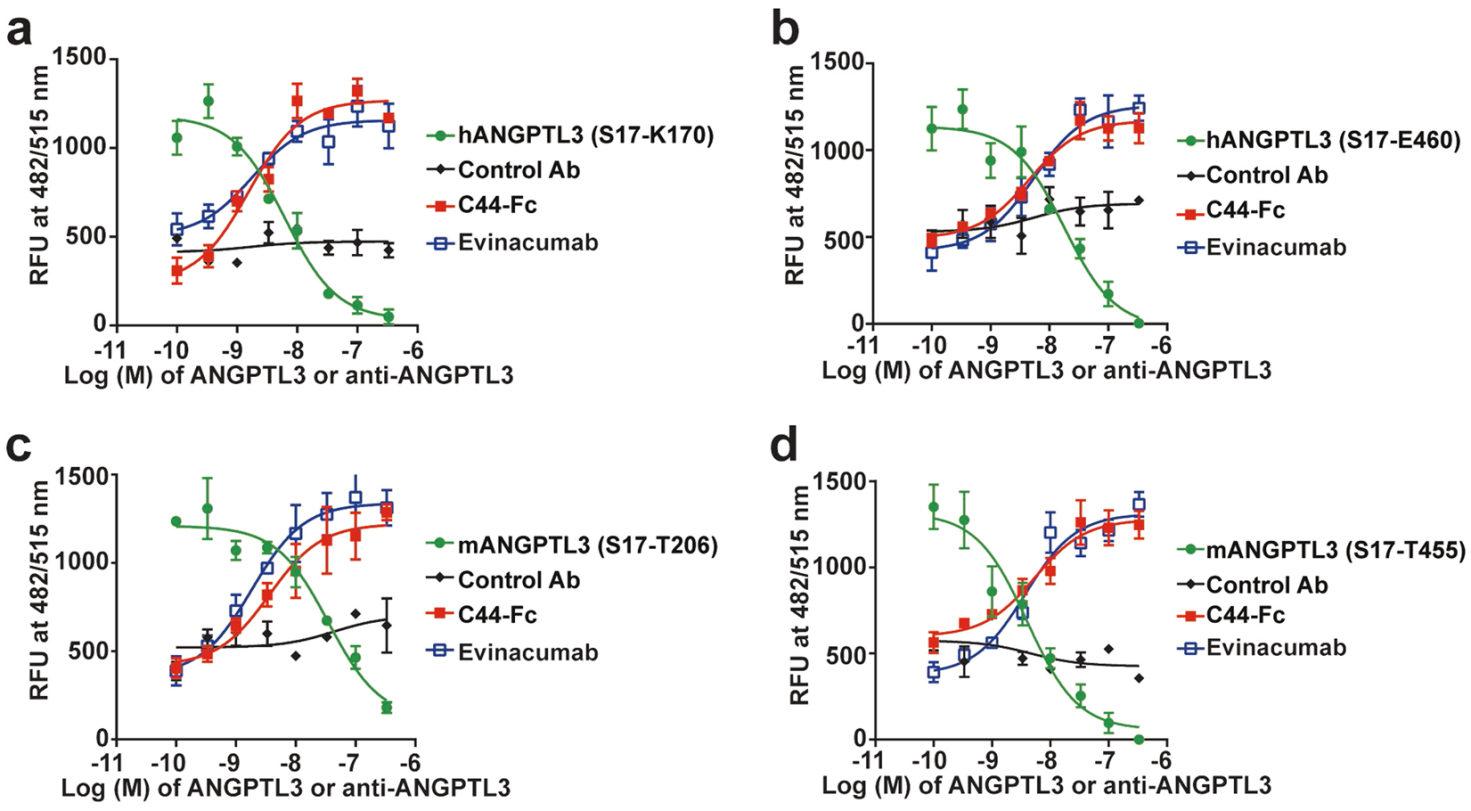
C44-Fc suppresses ANGPTL3-induced blockade of LPL. The vertical axis indicates the incremental concentrations of four kinds of ANGPTL3 proteins or antibody including of C44-Fc, evinacumab and control Ab (n = 3). The vertical axis indicates the relative fluorescence units (RFU) at 482/515 nm (excitation/emission)

We further studied the ability of C44-Fc to block ANGPTL3-mediated suppression of LPL activity by a cell-free LPL assay in vitro. The C44-Fc effectively neutralized the inhibition of LPL activity inducing by four different ANGPTL3 proteins (including CDD and full-length of human and mouse ANGPTL3) with IC_50_ values from 1.6 to 5.4 nM (Fig. 5 and Table 1). Because C44-Fc bound to human and mouse ANGPTL3 proteins with high affinities and efficiently blocked their inhibitory effect, we employed C57BL/6 mice to assess the following pharmacodynamics study in vivo*.*

**Table 1:** IC_50_ values for C44-Fc inhibition of ANGPTL3-induced LPL suppression

Parameter	Protein			
	hANGPTL3 (S17-K170)	hANGPTL3 (S17-E460)	mANGPTL3 (S17-T206)	mANGPTL3 (S17-T455)
C44-Fc, IC_50_ (nM)	1.6	4.6	3.6	5.4
Evinacumab, IC_50_ (nM)	1.7	5.4	1.8	3.7
Control Ab, IC_50_ (nM)	NB	NB	NB	NB

The IC_50_ values were measured using constant concentrations of ANGPTL3 proteins that were less than 2.5-fold of the EC_50_ values for ANGPTL3-induced suppression of LPL

### C44-Fc administration lowered serum lipids levels in hypercholesterolemic mice

**Fig. 6:**
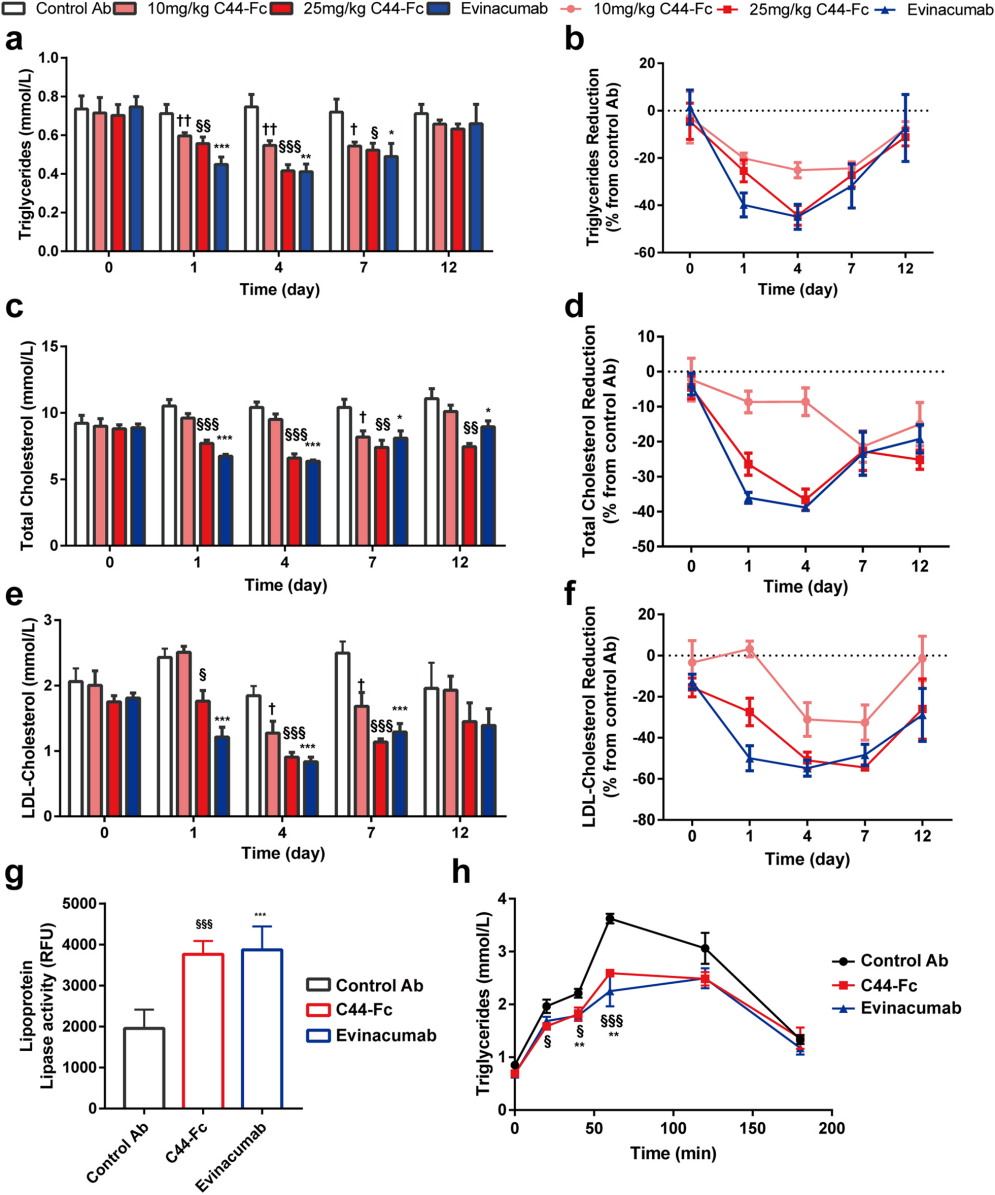
C44-Fc reduces serum TG, TC and LDL-C levels in hypercholesterolemic mice. C57BL/6 mice were induced by HF/HCCD-fed for 4 weeks to establish hypercholesterolemic mice model and then treated with isotype control Ab (25 mg/kg), C44-Fc (10 mg/kg or 25 mg/kg) and evinacumab (25 mg/kg). Serum TG (**a**), TC (**c**) and LDL-C (**e**) levels were tested enzymatically (n = 6–8). The TG (**b**), TC (**d**) and LDL-C (**f**) reduction levels of C44-Fc or evinacumab-treated group from control Ab-treated group were also measured. **g** Postheparin plasma LPL activities of HF/HCCD-fed mice treated with control Ab, C44-Fc and evinacumab (n = 6). **h** Lipid tolerance test were performed 4 days after control Ab, C44-Fc and evinacumab treatment (n = 6). †, § and * respectively represent the Student’s t-test of 10 mg/kg C44-Fc, 25 mg/kg C44-Fc and 25 mg/kg evinacumab treatment compared with control Ab

C57BL/6 mice were fed with a high-fat/high-cholesterol and cholate diet (HF/HCCD) for 4 weeks to induce hypercholesterolemic model and assess the pharmacodynamics of C44-Fc in vivo. Mice were then grouped randomly and treated with a single dose of isotype control antibody (control Ab, 25 mg/kg), C44-Fc (10 mg/kg or 25 mg/kg), or evinacumab (25 mg/kg). Blood samples were acquired on days 0, 1, 4, 7 and 12 after antibody administration. C44-Fc and evinacumab evoked rapid downward trends in serum TG, TC, and LDL-C levels. On day 4, C44-Fc respectively decreased the TG and TC level by 44.2% and 36.6% when compared with isotype controls (Fig. 6a–d). While evinacumab showed similar reduction effects in TG and TC level (44.9% and 38.9%, respectively). During day 4 ~ 7 post antibody administration, LDL-C levels in C44-Fc and evinacumab-treated mice were significantly reduced over 54.4% (Fig. 6e, f). Furthermore, the hypercholesterolemic mice receiving a single dose of C44-Fc or evinacumab still exhibited considerably lower TC level on day 12 (the end point of in vivo assay).

We further confirmed the LPL activity in hypercholesterolemic mice after C44-Fc treatment. Our results showed that the effect of C44-Fc on the serum lipids reduction was closely related with the upregulated activity levels of plasma LPL activity after intraperitoneally injecting heparin (Fig. 6g). The facilitation of TG clearance triggered by C44-Fc was further assessed by fat tolerance tests. After injection of intralipid, plasma TG of mice treated with control Ab had a remarkable increase which was significantly attenuated by C44-Fc (Fig. 6h), indicating C44-Fc administration remarkably improved fat tolerance in HF/HCCD-fed mice.

### C44-Fc administration relieved HF/HCCD-induced hepatic steatosis

**Fig. 7:**
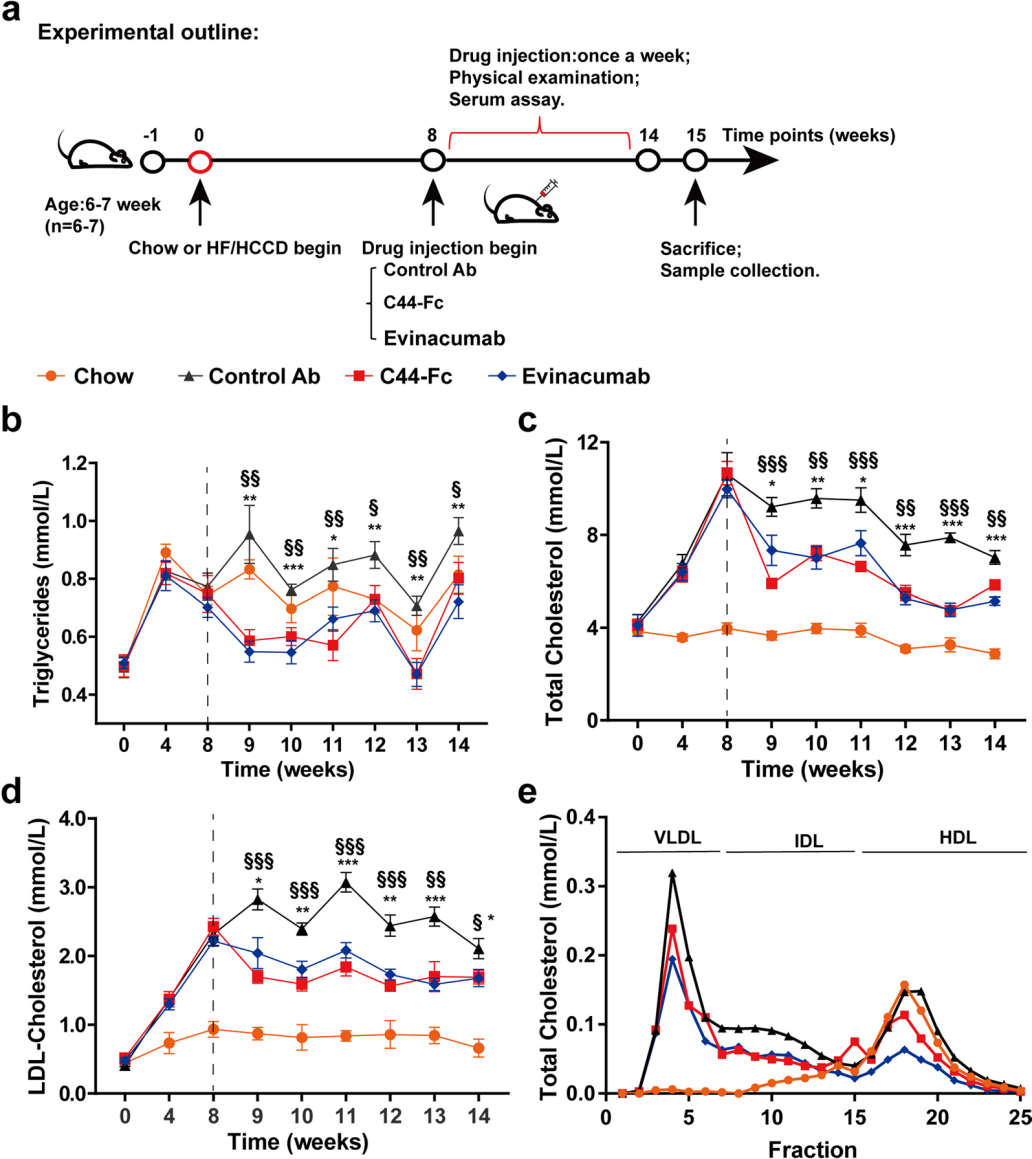
C44-Fc administrations reduce serum lipid levels for 6 weeks. **a** Schematic illustration of experimental design to assess the therapeutic effects of C44-Fc in NAFLD mice after multiple administrations. NAFLD model in C57BL/6 mice were induced by HF/HCCD-fed for 8 weeks and then treated with 25 mg/kg of isotype control Ab, 25 mg/kg of C44-Fc, or 25 mg/kg of evinacumab weekly. Serum TG (**b**), TC (**c**) and LDL-C (**d**) levels were weekly tested 4 days after antibody administrations (n = 6). Plasma lipoproteins of cholesterol (**e**) were separated by HPLC and measured in each fraction. § and * respectively represent the Student’s t-test of 25 mg/kg C44-Fc and 25 mg/kg evinacumab treatment compared with control Ab

The therapeutic effect of C44-Fc on NAFLD was investigated in HF/HCCD-fed mice. After 8 weeks after HF/HCCD feeding, C57BL/6 mice showed the marked increase in plasma TC and LDL-C levels from 4.3 to 10.6 mmol/L and from 0.41 to 2.3 mmol/L, respectively. Then mice were continuously dosed with isotype control Ab (25 mg/kg), C44-Fc (25 mg/kg), or evinacumab (25 mg/kg) once a week for 6 weeks (Fig. 7a). Blood samples were collected after 4-h fasting on day 4 after each injection. Compared with the chow-diet mice, HF/HCCD mice had increase in liver weight (~ 1.7-fold), liver TG contents (~ 1.6-fold) and hepatic lipid accumulation, indicating the pathological changes of NAFLD in HF/HCCD mice [36, 37]. The treatments with C44-Fc (red lines) or evinacumab (blue lines) decreased plasma TG, TC and LDL-C markedly and sustainably in comparison to isotype control Ab (Fig. 7b–e). After the first injection of C44-Fc, a significant decline trends in TG (from 0.95 to 0.59 mmol/L), TC (from 9.2 to 5.9 mmol/L) and LDL-C (from 2.8 to 1.7 mmol/L) levels were observed after 1 day post C44-Fc administration. Similarly, there were also noticeable reductions in TG (from 0.95 to 0.55 mmol/L), TC (from 9.2 to 7.3 mmol/L) and LDL-C (from 2.0 to 2.0 mmol/L) levels after the first administration of evinacumab.

**Fig. 8:**
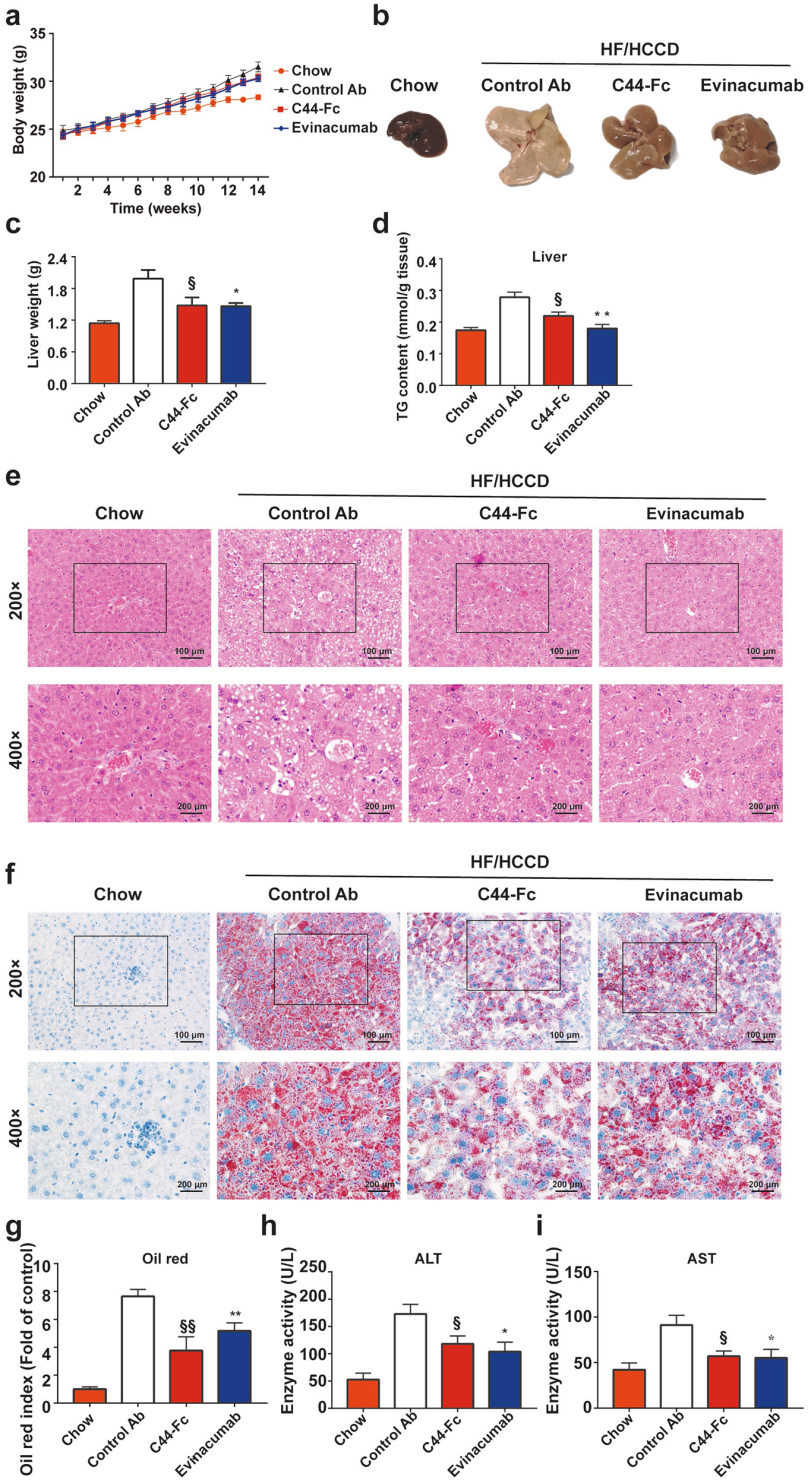
C44-Fc treatment relieves hepatic steatosis induced by HF/HCCD feeding. Body weight (**a**), representative liver morphology (**b**) and liver weight (**c**) of mice were presented (n = 6). **d** The TG contents of livers were measured (n = 5) by commercial kits. **e** Representative H&E staining of liver tissues. **f** Representative images and quantification (**g**) of oil red O staining are presented (n = 3). The levels of Serum ALT (**h**) and AST (**i**) were measured at the terminus of the research (n = 6). § and * respectively represent the Student’s t-test of 25 mg/kg of C44-Fc and 25 mg/kg of evinacumab treatment compared with control Ab

As shown in Fig. 8b, c, the liver overweight, liver coloration and morphology of HF/HCCD mice were significantly ameliorated after administration of C44-Fc for 6 weeks. Furthermore, our results also demonstrated that HF/HCCD feeding led to a hepatic lipid accumulation as well as increased level of ALT and AST, which were distinctly ameliorated by weekly dosage of C44-Fc (Fig. 8d–i). Fairly, 25 mg/kg of C44-Fc treatment had no significant influence on body weight and TG contents of epididymal fat or heart, insulin tolerance test (ITT) and glucose tolerance test (GTT) (Additional file 1: Figs. S3 and S4). Collectively, these findings suggested that C44-Fc fusion protein treatment efficiently reduced the serum lipids levels and relieved the hepatic steatosis induced by HF/HCCD feeding.

## Discussion

NAFLD is a metabolic disease and positively associated with hypercholesterolemia, which is increasingly acknowledged as the key factor of excessive lipid uptake and lipotoxicity in liver, resulting in pathologic insult [3]. It is projected to develop into cirrhosis with increasing risk of hepatocellular carcinoma and may be the main inducement of liver transplantation [4]. Despite its prevalence and seriousness, NAFLD still lacks of effective therapeutics [38]. ANGPTL3 is a key lipid metabolism regulator leading to high levels of plasma TG, TC and LDL-C [10]. In this study, we investigated whether inhibiting ANGPTL3 by VHH-Fc was an effective approach for NAFLD treatment. Herein, we developed a VHH-Fc fusion protein against ANGPTL3, which significantly ameliorated hepatic lipid accumulation and liver injury in HF/HCCD-induced NAFLD mice through reducing serum lipid levels of TG, TC and LDL-C.

Although NAFLD could be treated by reducing blood lipids and blood glucose, anti-fibrosis or anti-inflammatory in animal models, it still affects approximately 25% of the population worldwide [39]. Until now, the PPAR-α/γ agonist saroglitazar was exclusively approved for the treatment of NAFLD [7]. Unfortunately, the PPAR-α/γ agonist had been reported to increase about 2–4% of body weight and had adverse reactions including peripheral oedema in probably 5% of treated patients and fatal heart failure in approximately 11% of treated patients [40]. The classical lipids-reducing agents statins were not applicable to NAFLD as for its intolerance or side effects [41]. Thus, safe and efficient targets for NAFLD are urgently needed. ANGPTL3, as an inhibitor of LPL and EL, was recently revealed elevated expression levels in liver of NAFLD patients and contributed to increased circulating ANGPTL3 [16]. Interestingly, neither the homozygous loss-of-function of ANGPTL3 nor the inhibitors of ANGPTL3 did generate adverse effects with preeminent safety and tolerability, but leaded to very low TG, VLDL, LDL and non-esterified fatty acids (NEFAs) [42, 43]. Our results suggested ANGPTL3 inhibitor C44-Fc significantly reduced TG (44.2%), TC (36.6%) and LDL-C (54.4%). More importantly, multiple administrations of C44-Fc significantly reduced 18.3% of liver weight, ameliorated liver coloration and morphology, improved hepatic lipid accumulation and protected HF/HCCD-related liver injury. All these results indicated that blocking ANGPTL3 was a novel therapeutic approaches for NAFLD with satisfactory efficacy and safety (in Additional file 1: Fig. S5).

Nanobody and its fusion protein, which are promising therapeutic agents, display excellent binding affinity and stability. Besides, they could be expressed in yeast with high yield and low cost [44]. Recently, a bivalent VHHs fusion protein, caplacizumab, had been approved by the FDA for thrombotic thrombocytopenic purpura treatment, indicating the druggability of nanobody and its fusion protein [35, 45, 46]. The development and characterization of VHHs that specifically block ANGPTL3 have not been reported so far. Hence, we established an immune phage-display VHHs library after immunizing alpaca with hANGPTL3 (S17-K170) and obtained 3 specific VHH candidates which had different CDR3 sequences from the library. Finally, we retrieved C44 clone for in-depth study that had the highest affinity to the CCD of hANGPTL3 at 0.6026 nM.

The VHHs have prominently short circulation half-life due to their small size of 15 kDa. Multiple methods have been applied to increase their half-life period in vivo, such as binding to human serum albumin, fused to IgG-Fc and PEGylated [47, 48]. Meanwhile, previous studies revealed that bivalent nanobody had better affinity than monovalent modality. In particular, the bivalent form via Fc fusion showed above 10 times higher affinity and stronger therapeutic effects than monovalent in vitro and in vivo, also emerged longer half-life up to 15 days when compared with monovalent (~ 30 min) and tandem bivalent VHHs (~ 60 min) [44, 49]. In present study, C44 was fused to human IgG1 Fc domain to construct the bivalent VHH-Fc fusion protein. The affinities of C44-Fc to ANGPTL3 proteins were about 2.1–11.0 times higher than the individual C44. The pharmacodynamics study of single administration demonstrated that the C44-Fc fusion protein had a long half-life in vivo and the durable lipid-reducing effect lasted up to 12 days.

When compared to the conventional antibody evinacumab, C44-Fc fusion protein had similar ability of rescuing ANGPTL3-mediated suppression of LPL activity and the lipid-reducing effect as well as relieving function in NAFLD. However, the yield of camelidae antibody C44-Fc in ExpiCHO cells reached ~ 500 mg/L, which was twice as much as evinacumab We also investigated the stability of C44-Fc at pH 5, pH 9, 40 °C for 28 days and 6 cycles of freeze–thaw. The results proved that C44-Fc fusion protein showed satisfying stability with no obvious degradation or aggregation at these conditions, indicating that C44-Fc might decrease the expenditure of production, storage and transportation in the future development as a therapeutic antibody.

Consistent with these results, C44-Fc, as an ANGPTL3 inhibitor, showed remarkable potency in lowering lipid and therapeutic effect on NAFLD in mice model, and was likely to be a prospective therapeutic agent for hypercholesterolemic and NAFLD patients. It also might be a latent treatment in other metabolic disorders such as cardiovascular diseases. Nonetheless, further research such as humanization, pharmacodynamics study in cynomolgus monkeys and optimization of expression system, should be conducted to develop it into a therapeutic agent.

## Conclusions

In this work, we demonstrated that blocking ANGPTL3 by VHH-Fc fusion protein could relieve hepatic lipid accumulation and liver injury in HF/HCCD-induced NAFLD mice. We developed a novel VHH-Fc fusion protein (C44-Fc) that showed superior characteristics in affinity, yield and stability. We further illustrated its potency in inhibiting ANGPTL3-mediated LPL activity in vitro, lipid-reducing function in hypercholesterolemic mice, as well as ameliorating efficacy in NAFLD mice, indicating that the C44-Fc could be a potent therapeutic candidate for NAFLD.

## Methods

### Immunization and library generation

Alpaca immunizations and the following construction of VHH library were performed as reported previously [50]. A female alpaca was subcutaneously injected with 500 μg of emulsified hANGPTL3(17-170)-mFc antigen (fused from human S17-K170 ANGPTL3 and mouse IgG1-Fc and expressed by our laboratory) on days 1, 21, 42, 63. Blood was collected at 7 days after the last immunizations for the isolation of peripheral blood lymphocytes (PBLs) by density gradient centrifugation using ficoll-paque plus (Cytiva, USA, Cat# 17144002). The total RNA of PBLs was extracted by Trizol, followed by a RT-PCR for amplification of the coding sequences of VHHs. Next, the VHHs library was generated by electro-transforming the VHH genes and pHEN1 phagemid recombinant vector into competent *E.coli* TG1. Finally, the VHHs library was rescued by coinfection with M13KO7 helper phages (Thermo Scientific Ltd, USA, Cat# 18311019).

### hANGPTL3-specific VHHs enrichment

Phages presenting VHHs with hANGPTL3-specific binding were enriched after three rounds of screening on hANGPTL3 (S17-220P)-His-immobilized (Novoprotein, China, Cat# Q9Y5C1) 96-well plates. The concentrations of antigen coated on every well for three rounds were decreased in the order of 5, 2, 1 μg per well and simultaneously a blank well was employed as a negative control. After washing with PBST for three times, all wells were blocked in a 37 °C constant temperature incubator for 1 h with PBS containing 3% (w/v) milk powder and washed with PBS for 3 times. Approximately 10^11^ phages (diluted with PBS containing 3% (w/v) milk powder) were added to the antigen-coated plates and then put into a 37 °C constant temperature incubator for 1 h. After washing with PBST (containing 0.05% Tween 20) for 6 times in the first round and 8 times in the remaining two panning rounds, hANGPTL3-specific phages were retained and added into *E. coli* TG1culture to amplify at 30 °C with shaking and rescued with M13KO7 helper phages at 30 °C overnight. The purified phages were precipitated by PEG 8000/NaCl for the subsequent panning round. For each bio-panning, enrichment was performed as described above and was evaluated by the bacteria population which were infected with the purified phages and cultured on LB agar plates with ampicillin.

### Indirect ELISA screening

ELISA was employed to evaluate the affinity of phage-displayed VHH to ANGPTL3. Forty-eight individual bacteriophages were selected from the second and third bio-panning LB agar plates and rescued with M13KO7 helper phages at 30 °C overnight. Next, the viral supernatants containing VHHs were added into the plates coated with hANGPTL3 (S17-220P)-His protein, and PBS containing 3% (w/v) milk powder was used as a negative control. After washing with PBST (containing 0.05% Tween 20) for 6 times, the bound VHHs were determined by HRP-conjugated rabbit anti-M13 antibody (1:500, Abcam, USA) that could react with TMB (Beyotime, China, P0209) followed by the OD450 value testing. Finally, the bacteriophages with OD450 value five-fold higher than the negative control were regarded as positive clones and used for subsequent sequencing.

### Nanobody expression and purification

The C27, C44 and C46 gene sequences with 6 × His tag at the amino terminus were synthesized and cloned into the pET21a expression vectors, that were then transformed into *E. coli* Origami B(DE3) for the nanobody expression. These *E. coli* Origami B(DE3) cells were inoculated into a LB medium with 100 µg/mL of ampicillin at 37 °C, 220 rpm. The cells were induced by 0.1 M IPTG (Sangon Biotech, China) when they reached the logarithmic growth phase and cultured at 220 rpm at 28 °C for 16 h. The nanobodies were harvested from the cytoplasm of *E. coli* Origami B(DE3) cells by ultrasonic degradation. Finally, the nanobodies were purified by HisTrap IMAC HP column (GE Healthcare, China).

### Epitope binning test

The 100 ng/well of evinacumab was coated on the ELISA plate at 4 °C for 12 h. After washing the ELISA plate with PBST and blocking with 3% BSA, the 1 μg/well of hANGPTL3 (S17-K170)-Fc antigen was added for two hours at 25 °C. After washing the ELISA plate with PBST, the C44-His6 was then added to bind the other epitopes on the hANGPTL3 (S17-K170)-Fc for 2 h at 25 °C. After washing the ELISA plate with PBST for three times, the bound C44-His6 were determined by the His tag polyclonal antibody with HRP (1:10,000 dilution, Thermo Scientific Ltd, USA) that could react with TMB (Beyotime, China, P0209) followed by the OD450 value testing.

### VHHs-Fc expression and purification

The encoding sequence of C44 fused to human IgG1-Fc (with the hinge region and had mutated the first Cys into Ser) was synthesized and inserted into pTT5 vector. The DNA sequences encoding the variable regions of evinacumab were cloned and digested into corresponding regions of human IgG1 on pTT5 vector by EcoRI and NheI (Takara, Biotechnology, Otsu, Japan). The C44-Fc and evinacumab expression plasmids were transfected into ExpiCHO-S cells (Gibco, Thermo Scientific Ltd.) by CHO Transfection Kit and cultured in ExpiCHO expression media (Gibco, Thermo Scientific Ltd.) according to its Max Titer protocol. The culture supernatants were collected 12 days after transfection and purified by HiTrap MabSelect Prism A column (GE Healthcare).

### Affinity determination

The affinities of antibody binding to hANGPTL3 (S17-P220)-His6, hANGPTL3 (S17-E460)-His10, mANGPTL3 (S17-T206)-His6 and mANGPTL3 (S17-T455)-His10 were determined by SPR on a Biacore T200 system (GE Healthcare, USA). The antigen hANGPTL3 (S17-220P)-His, hANGPTL3 (S17-E460)-His10, mANGPTL3 (S17-T206)-His6 and mANGPTL3 (S17-T455)-His10 were immobilized on the CM5 chip and then antibodies were flowed over the chip at 120 s for binding and 480 s for dissociation after dilution by EP buffer. The regeneration buffer of pH 2.5 Glycine–HCl (GE Healthcare, USA) was flowed over the chip for 30 s before the next round. The affinity (KD) was calculated by Biacore Evaluation software as followings: KD (nM) = kd (1/s)/ka (1/Ms). kd is the dissociation constant, ka is the binding constant.

### Biophysical stability evaluation

SEC-HPLC was performed to assess the concentration of VHHs-Fc using an Agilent 1260 Infinity II SFC System with TOSOH TSKgel G3000WXL column (7.8 mm × 30 cm, 5 μm) at 1.0 mL/min flow rate of PBS. Each sample was injected with 100 μg of protein and measured by UV detection for 30 min at 280 nm and 37 °C.

### In vitro LPL assay

The ability of C44-Fc to block ANGPTL3-mediated suppression of LPL activity was tested by a cell-free LPL assay in vitro. Four kinds of ANGPTL3 proteins included hANGPTL3 (S17-K170)-mFc, hANGPTL3 (S17-E460)-His10, mANGPTL3 (S17-T206)-His6 and mANGPTL3 (S17-T455)-His10 or with C44-Fc, evinacumab or control antibody (Ab) were pre-incubated with 40 nM of bovine LPL (Sigma, USA, 9004-02-8) and 0.46 µM of human ApoCII (EMD Biosciences, San Diego, CA), and then were measured by LPL Activity Fluorometric Assay Kit (Biovision, USA) at 37 °C for 1 h. Fluorescence was tested on a BioTek Multi-Mode Microplate Reader at 482/515 nm (excitation/emission).

### Animal study

C57BL/6 mice (male, 5–6 weeks old) were obtained from Shanghai SIPPR-BK Laboratory Animal Co., Ltd. (Shanghai, China) and were kept under the standard specific pathogen-free (SPF) environment. Mice were supplied with high-fat/high-cholesterol and cholate diet (HF/HCCD, Teklad TD.90221, which contains 15.8% fat, 1.25% cholesterol and 0.5% sodium cholate) or chow diet after being acclimated to experiment environment for 1 week [51]. For single administration studies, C57BL/6 mice were fed with HF/HCCD for 4 weeks and then treated with subcutaneous injection of control Ab, C44-Fc or evinacumab. Blood samples were collected before antibody injection as a baseline and 1, 4, 7 and 12 days later by retro-orbital bleed after a 4 h fast. For the postheparin plasma LPL activity, mice were treated with heparin (100 U/kg) intraperitoneally injected in tail and 5 min later plasma samples were collected into EDTA-coated tubes which were preserved on ice. As for multiple administration, C57BL/6 mice were fed with HF/HCCD for 8 weeks and treated with subcutaneous injection of control Ab, C44-Fc or evinacumab weekly for 6 weeks. Blood samples were collected 4 days after each injection. At the end of the experiment, plasma lipoproteins were separated as described previously [52].

### Metabolic analysis

Lipid tolerance test (LTT), insulin tolerance test (ITT), and glucose tolerance test (GTT) were performed 4 days after antibody injection (25 mg/kg). For LTT, C57BL/6 mice were injected intraperitoneally with 10 μg/kg body weight of intralipid (Baxter Healthcare Corporation, USA) after a 2-h fast. For ITT, mice were intraperitoneally injected with 1 IU insulin (Beyotime, Shanghai) per kg body weight after a 4-h fast. For GTT, mice were intraperitoneally injected with 1 g/kg body weight of glucose (Beyotime, Shanghai) after 6 h of fasting. All blood samples were acquired at indicated time points after C44-Fc administration.

### Biochemistry analysis

All serum levels of TG, TC, LDL-C, alanine aminotransferase (ALT), aspartate aminotransferase (AST) and tissue lipid content were detected using commercial kits (Nanjing Jiancheng Bioengineering, Nanjing). The blood glucose levels were tested by blood glucose test strips (Roche, Germany).

### Histopathological study

The liver samples were weighed and fixed with 4% paraformaldehyde. Subsequently, all liver samples were embedded with paraffin and stained with hematoxylin and eosin (H&E) or embedded in OTC and freezed for oil red O staining.

### Data analyses

All data were presented as mean ± SEM. The significant difference between groups were operated using Student’s t-test or one-way ANOVA and were indicated †, § or * as P < 0.05, ††, §§ or ** as P < 0.01 and §§§ or *** as P < 0.001.

## Supplementary Information

**Additional file 1: Figure S1.** ELISA assay of plasmatic antibody titer from pre-immune and immunized alpaca (n = 3). The value ratio of post/pre-immune plasmatic ELISA ≥ 2.1 is recorded as positive (*). **Figure S2.** Evaluation the insertion rate of correct VHH clones in library. The PCR products of positive clones inserted VHH genes were about 700 bp. **Figure S3.** The weight (**A**) and TG content (**B**) of white adipose tissue (epiWAT) and hearts (n = 5). All tissue were acquired at the end of multiple lipids-lowering treatments. **Figure S4.** Insulin tolerance test (ITT, **A**) and glucose tolerance test (GTT, **B**) were performed at the 6th week of multiple lipids-lowering treatments (n = 5). **Figure S5.** Long-term C44-Fc injection did not manifest systemic toxicity in mice. H&E staining of major organs (heart, spleen, lung, kidney and brain) after 4-week treatment were presented.

## Data Availability

All data generated or analyzed during this study are included in the article.

## Abbreviations

ALT: Alanine aminotransferase
ANGPTL3: Angiopoietin-like protein 3
AST: Aspartate transaminase
ASO: Antisense oligonucleotide
CCD: Coiled-coil domain
EL: Endothelial lipase
ELISA: Enzyme-linked immunosorbent assay
Fc: The constant region fragment of the human immunoglobulin gamma
FH: Familial hypercholesterolemia
FLD: Fibrinogen-like domain
GTT: Glucose tolerance test
H&E: Hematoxylin and eosin
HF/HCCD: High-fat/high-cholesterol and cholate diet
HRP: Horse radish peroxidase
IgG: Immunoglobulin gamma
ITT: Insulin tolerance test
LDL: Low density lipoprotein
LDL-C: Low density lipoprotein cholesterol
LDLR: Low density lipoprotein receptor
LPL: Lipoprotein lipase
LTT: Lipid tolerance test
LXRs: Liver X receptors
OD450: The optical density value at 450 nm
PBLs: Peripheral blood lymphocytes
PBST: Phosphate buffer solution with 0.05% tween 20
NAFLD: Non-alcoholic fatty liver disease
RFU: The relative fluorescence unit
RU: Response units
sdAb: Single domain antibody
SPR: Surface plasmon resonance
TC: Total cholesterol
TES buffer: 0.2 M Tris–HCl pH 8.0: 0.5 mM EDTA: 0.5 M sucrose
TG: Triglyceride
VHH: High variable region of the heavy chain antibody
kDa: Kilo Dalton
