# Supplementary material for: A novel nanobody-heavy chain antibody against Angiopoietin-like protein 3 reduces plasma lipids and relieves nonalcoholic fatty liver disease
Source: J Nanobiotechnology. 2022 May 19;20:237. doi: 10.1186/s12951-022-01456-z (PMC9118633; doi:10.1186/s12951-022-01456-z)
Supplement: Supplementary file 1 — Additional file 1: Figure S1. ELISA assay of plasmatic antibody titer from pre-immune and immunized alpaca (n = 3). The value ratio of post/pre-immune plasmatic ELISA ≥ 2.1 is recorded as positive (*). Figure S2. Evaluation the insertion rate of correct VHH clones in library. The PCR products of positive clones inserted VHH genes were about 700 bp. Figure S3. The weight (A) and TG content (B) of white adipose tissue (epiWAT) and hearts (n = 5). All tissue were acquired at the end of multiple lipids-lowering treatments. Figure S4. Insulin tolerance test (ITT, A) and glucose tolerance test (GTT, B) were performed at the 6th week of multiple lipids-lowering treatments (n = 5). Figure S5. Long-term C44-Fc injection did not manifest systemic toxicity in mice. H&E staining of major organs (heart, spleen, lung, kidney and brain) after 4-week treatment were presented. [file 12951_2022_1456_MOESM1_ESM.docx]

**A novel nanobody-heavy chain antibody against Angiopoietin-like protein 3 reduces plasma lipids and relieves nonalcoholic fatty liver disease**

Xiaozhi Hu^1,#^, Jiajun Fan^1,#^, Qianqian Ma^1,2,#^, Lei Han^1,#^, Zhonglian Cao^1^, Caili Xu^1^, Jingyun Luan^1,3^, Guangjun Jing^1^, Yanyang Nan^1^, Tao Wu^1^, Yuting Zhang^1^, Hanqi Wang^1^, Yuanzhen Zhang^1^, Dianwe Ju^1,*^

#These authors contributed equally to this work.

*Correspondence to: Prof. Dianwen Ju, E-mail: dianwenju@fudan.edu.cn

School of Pharmacy & Minhang Hospital, Shanghai Engineering Research Center of Immunotherapeutics, Fudan University, Shanghai, 201203, China


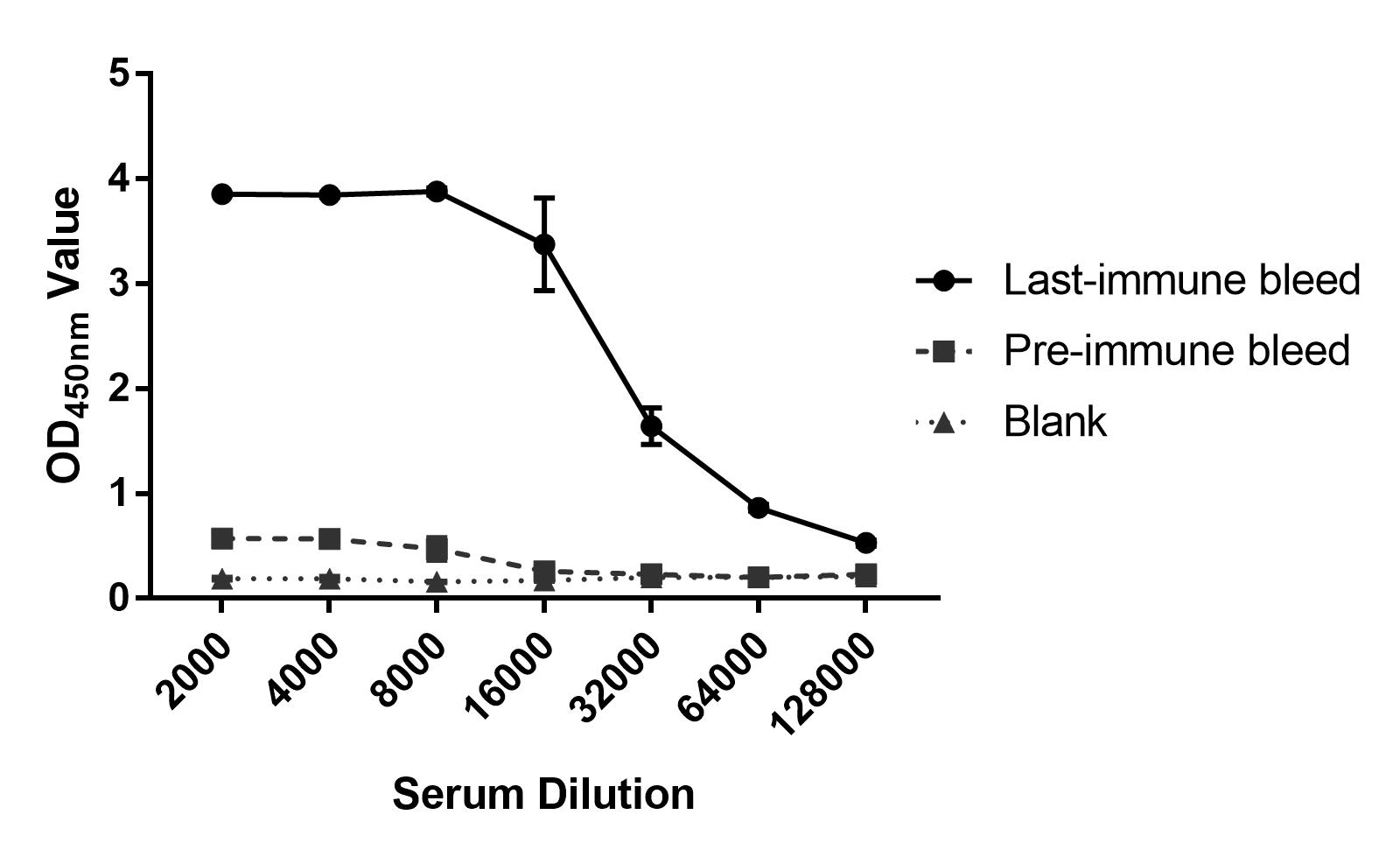


**Figure S1** ELISA assay of plasmatic antibody titer from pre-immune and immunized alpaca (n = 3). The value ratio of post / pre-immune plasmatic ELISA ≥ 2.1 is recorded as positive (*)


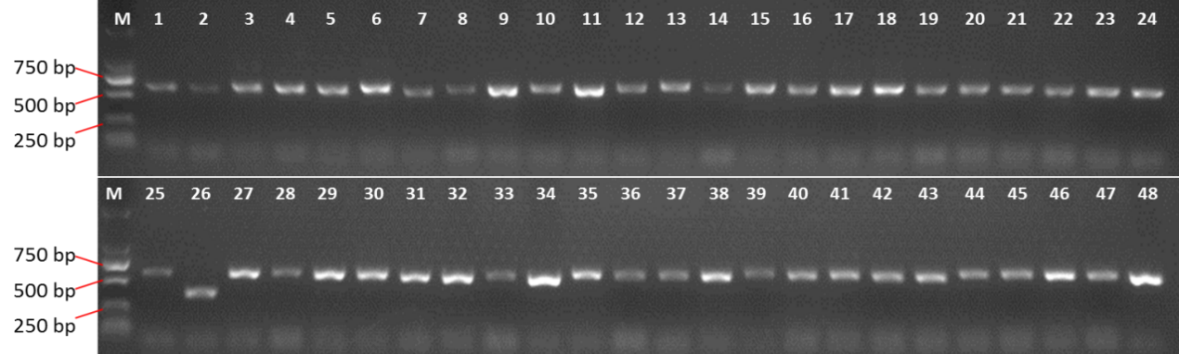


**Figure S2** Evaluation the insertion rate of correct VHH clones in library. The PCR products of positive clones inserted VHH genes were about 700 bp.


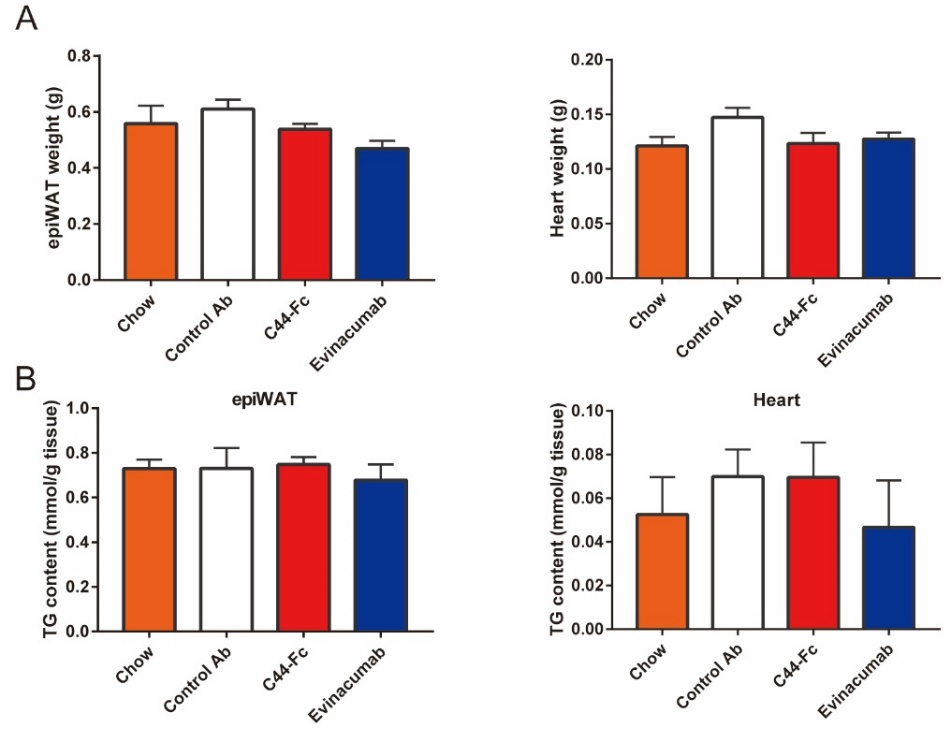


**Figure S3** The weight (A) and TG content (B) of white adipose tissue (epiWAT) and hearts (n = 5). All tissue were acquired at the end of multiple lipids-lowering treatments.


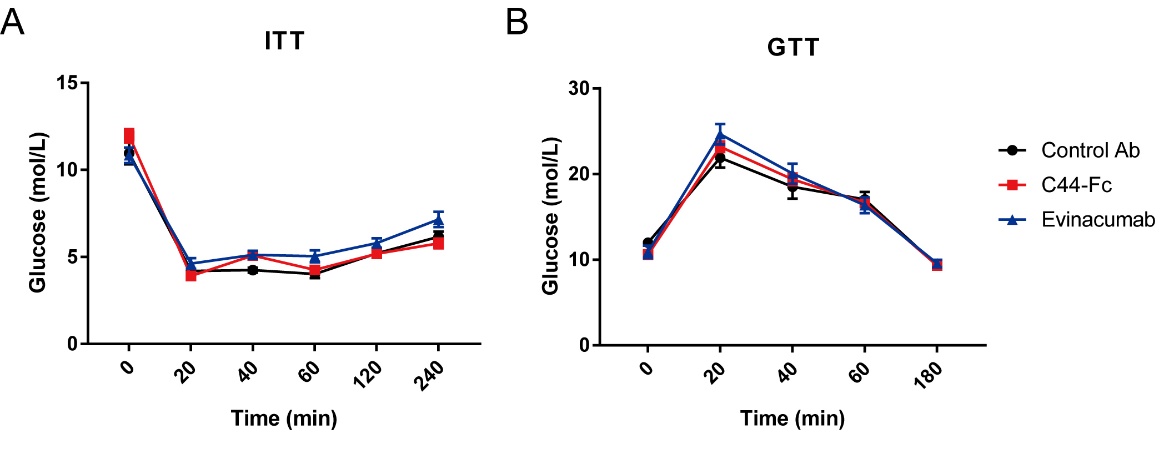


**Figure S4** Insulin tolerance test (ITT, A) and glucose tolerance test (GTT, B) were performed at the sixth week of multiple lipids-lowering treatments (n = 5).


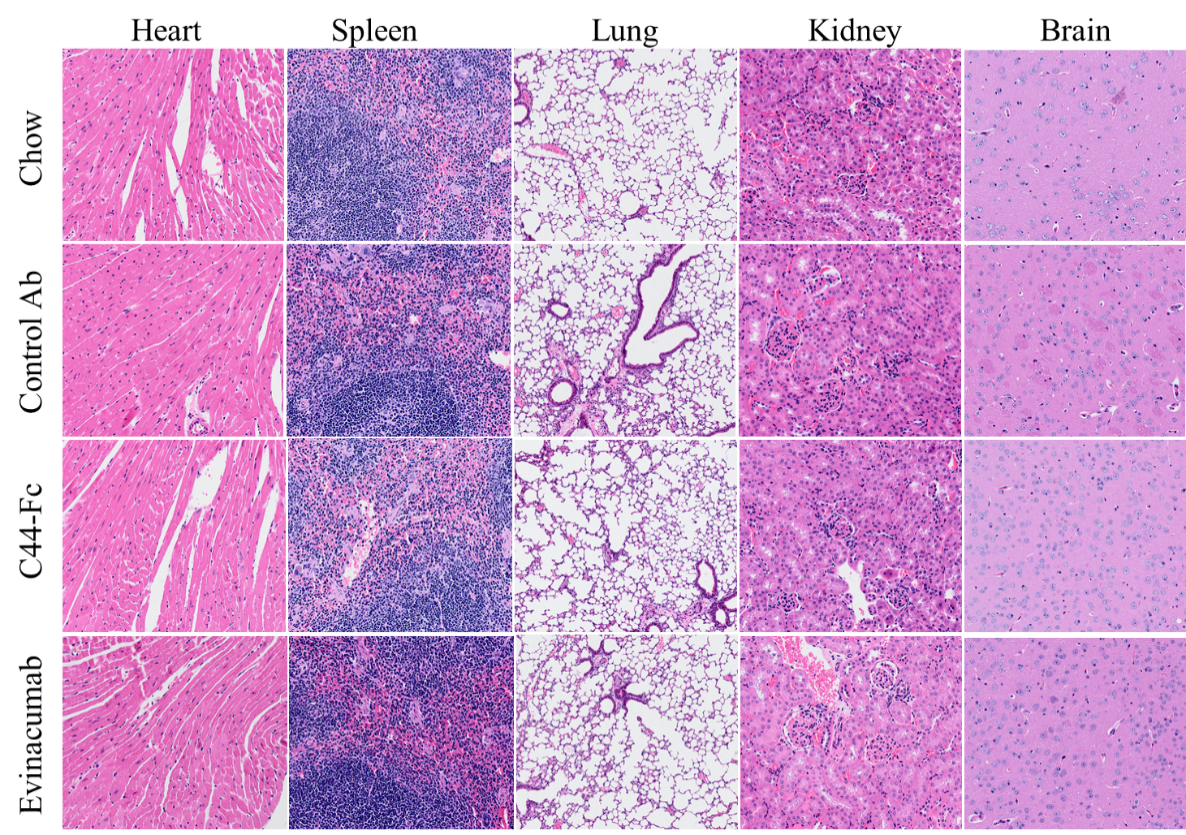


**Figure S5** Long-term C44-Fc injection did not manifest systemic toxicity in mice. H&E staining of major organs (heart, spleen, lung, kidney and brain) after 4-week treatment were presented.
